# Notoginsenoside R1 Protects Against the Acrylamide-Induced Neurotoxicity *via* Upregulating Trx-1-Mediated ITGAV Expression: Involvement of Autophagy

**DOI:** 10.3389/fphar.2020.559046

**Published:** 2020-09-02

**Authors:** Wenjun Wang, Lu Huang, Elizabeth Rosalind Thomas, Yingying Hu, Fancai Zeng, Xiang Li

**Affiliations:** ^1^Department of Biochemistry and Molecular Biology, School of Basic Medical Science, Southwest Medical University, Luzhou, China; ^2^Institute for Cancer Medicine and School of Basic Medical Sciences, Southwest Medical University, Luzhou, China; ^3^Department of Biotechnology and Bioinformatics, North Eastern Hill University, Shillong, India

**Keywords:** acrylamide, notoginsenoside r1, thioredoxin-1, autophagy, integrin alpha V

## Abstract

Acrylamide (ACR) is a common chemical used in various industries and it said to have chronic neurotoxic effects. It is produced during tobacco smoking and is also generated in high-starch foods during heat processing. Notoginsenoside R1 (NR1) is a traditional Chinese medicine, which is used to improve the blood circulation and clotting. The objective of this study was to investigate the mechanism of ACR-triggered neurotoxicity and to identify the protective role of NR1 by upregulating thioredoxin-1 (Trx-1). Our results have shown that NR1 could block the spatial and cognitive impairment caused by ACR administration. Bioinformatics analysis revealed that Trx-1 regulated autophagy *via* Integrin alpha V (ITGAV). NR1 could resist the ACR-induced neurotoxicity by upregulating thioredoxin-1 in PC12 cells and mice. The autophagy-related proteins like autophagy-related gene (ATG) 4B, Cathepsin D, LC3 II, lysosomal-associated membrane protein 2a (LAMP2a), and ITGAV were restored to normal levels by NR1 treatment in both PC12 cells and mice. Besides, we also found that overexpression of Trx-1 resisted ACR-induced autophagy in PC12 cells and downregulation of Trx-1 triggered autophagy induced by ACR in PC12 cells. Therefore, it could be concluded that Trx-1 was involved in the autophagy pathway. Besides, we also found that ITGAV was an intermediate node linking Trx-1 and the autophagy pathway.

## Introduction

Acrylamide (ACR), a well-known water-soluble chemical, is extensively used in various industries. It is also an important reagent that is commonly used for laboratory research, for example, while performing gel electrophoretic separation of molecules. Because of its wide application, human beings are exposed to high levels of ACR (Adewale et al., 2015). Neurotoxicity is a typical phenomenon of ACR toxicity in animal and cell models. Some studies have proven that ACR toxicity could induce cogitative deficits by influencing the autophagic function of the neurons in the hippocampus (Hip) (Tan et al., 2019). Recently, it was found that ACR-induced cellular toxicity in neurons could lead to apoptosis (He et al., 2017), mitochondrial dysfunction (Zamani et al., 2017), and downregulate antioxidant signaling pathway (Pan et al., 2017). However, the relationship between ACR and autophagy in PC12 cells still remains unmapped.

Notoginsenoside R1 (NR1) is an efficient free radical scavenger. It has been known to possess antioxidant properties and can repress adhesion molecules and chemokines (Dou et al., 2012). NR1 is a conventional Chinese medicine used in the treatment of cardiovascular diseases (Chan et al., 2002; Chen et al., 2011) and acute ischemic stroke (Chen et al., 2008; He et al., 2011). Recent studies have shown that NR1 could reduce myocardial ischemia-reperfusion injury by the autophagy pathway (Liu X. W. et al., 2019), however, the detailed mechanism is not clear.

The responses of cells to reactive oxygen species (ROS) and nitric oxide synthase (NOS) can be changed through various mechanisms. ROS and autophagy are the key players that regulate cellular homeostasis in neural cells. Autophagy can be stimulated by ROS through different kinds of signaling pathways, and conversely, inhibit ROS-induced damage to cells and tissues. Li et al. (2015) proved that ROS can induce autophagy, however, it is also known that autophagy functions as a buffer system to maintain the ROS levels in the cells and reduce toxicity. Some studies have revealed that exposure to cadmium (2 μM) could extensively increase ROS production and induce autophagy. Autophagy induction was further proved by the upregulation of autophagy-related gene (ATG) 4 (Lv et al., 2018). In the other pathways, ATG4 could proteolyze pro-LC3 to form LC3 I. Later, LC3 I forms a conjugate with phosphatidylethanolamine (PE) by the transforming action of ATG7 and ATG3, thereby generating LC3 II. LC3 II then attaches to the autophagosome membrane triggering its elongation (Mondaca-Ruff et al., 2018). The lysosomal proteolytic enzyme Cathepsin D is the only aspartic-type protease that is ubiquitously found in every cell of the human body. It is expressed at high levels in the brain. Normally, Cathepsin D mediates proteolysis and is required for the neuronal cell homeostasis. This is acquired by the degeneration of the unfolded or oxidized protein compounds, which are moved to the lysosomes by the process of autophagy or endocytosis. Besides, previous studies have also reported that lysosomal-associated membrane protein 2 (LAMP2) was involved in the binding of phagosomes with lysosomes (Tanaka et al., 2000; Huynh et al., 2007) and thus it could contribute to the autophagy regulation.

Thioredoxin-1 (Trx-1) is known to have numerous biological applications, such as regulation of the cellular redox balance, activation of the different transcription factors, and protecting the neurons (Bai et al., 2003; Burkegaffney et al., 2005). Previous *in vivo* studies have shown that NR1 could enhance the expression of Trx-1 (Luo et al., 2011).

Integrin alpha V (ITGAV) heterodimers has been known to promote or suppress cancer development in epithelial tissues (Lee et al., 2018). Recent studies have proven that ITGAV was associated with epithelial-mesenchymal transition and cell migration (van der Horst et al., 2011; Shidal et al., 2018). However, there are very few studies which report the potential role of ITGAV in autophagy.

Although NR1 was said to possess protective properties against various neurological diseases, the mechanism behind this hypothesis remains unclear. Our previous study had shown that NR1 played a neuroprotective role in ACR-induced neurotoxicity by suppressing mitochondrial apoptosis (Wang et al., 2020). However, it is still unclear how Trx-1 affects apoptosis. In this study, by using bioinformatics analysis, our results have proven the neuroprotective properties of NR1 on ACR-induced autophagy in PC12 cells. More importantly, this study has revealed that NR1 could inhibit ACR-induced autophagy by increasing the expression of Trx-1 *via* regulation of ITGAV and suppression of autophagy. Therefore, our results showed that ITGAV was an intermediate node linking Trx-1 and autophagy pathway.

## Materials and Methods

### Animals

Male C57BL/6 mice (wild-type, weight 22–25 g, 8 weeks of age) were purchased from Chengdu Dossy Experimental Animals CO., LTD, China. All the mice were housed in plastic cages under controlled conditions: average temperature 23°C, 12 h light/dark cycles, and it had free access to food and water. The mice were randomly divided into four groups: control group (n=8), ACR group (n=8), NR1 group (n=8), and ACR+NR1 group (n=8). In the control group, the mice were administrated with saline (0.9%) once a day for 4 consecutive weeks. In the ACR group, the mice were administrated with ACR (20 mg/kg) once a day for 4 consecutive weeks. The dosage of ACR used in the experiment was based on previous reports (Santhanasabapathy et al., 2015). In the NR1 group, the mice were administrated with NR1 (25 mg/kg) once a day for 4 consecutive weeks. In the ACR+NR1 group, the mice were pretreated with NR1 for 30 min prior to ACR administration. After the behavioral test, the mice were sacrificed by cervical vertebra dislocation. The Hip was immediately dissected, frozen, and stored in a deep freezer at −80°C until the assays were performed. All procedures and protocols had been approved by the animal ethics council of Southwest Medical University and were in accordance with the National Institutes of Health Guide for the Care and Use of Animals.

### Morris Water Maze (MWM) Test

MWM was performed to evaluate the spatial learning and memory of the mice after ACR administration. This was performed according to previously published methods (Sharma et al., 2010). MWM consisted of a black circular pool (Height: 50 cm, Diameter: 120 cm) filled with water (Depth: 20 cm, temperature: 24 ± 2°C) and a circular platform (diameter: 10 cm) for animals to escape. The platform was 1 cm below the water surface. In order to monitor the mice during the experiment, a camera was installed right above the pool. The MWM was encircled with black and white extra-maze cues on the wall of the pool, which could enable the mice to easily identify the different quadrants. The procedure followed for performing the MWM test was as follows: on the first day, all the mice were allowed to swim freely in order to acclimatize to the new environment. During the next five days, the mice were trained four times a day and were introduced to the four water inlet points with their head facing the wall of the pool on the basis of quadrants I, II, III, and IV, respectively. If the mice found the platform (The platform was quadrant V) before the 60 s cut-off, it was allowed to stay on the platform for 5 s and then it was returned back to the home cage. If the mice didn’t find the platform in the pool, it was guided to the platform and assigned a latency of 60 s. After the maze test training, all the mice were dried off. For five consecutive days, each mouse were subjected to the four trials (inter-trial interval: 15–20 min). On the day of the maze test, a spatial probe test was performed to detect the spatial memory of mice. The speed of swimming of the mice in the pool was measured. Then, the platform was removed from the pool. The mice were released from the quadrant opposite to the target quadrant and were allowed to swim freely for 60 s. The number of times the mice crossed the quadrant V (which had earlier housed the platform) was recorded. The time taken for swimming in the target quadrant was recorded.

### Reagents

Antibody to Trx-1 (14999-1-AP, 1:1,000), antibody to β-actin (20536-1-AP, 1:1,000), antibody to ATG4B (15131-1-AP, 1:1,000), and antibody to Cathepsin D (21327-1-AP, 1:1,000) were purchased from ProteinTech (Wuhan, China). Antibody to LC3 (4108S, 1:1,000) was purchased from Cell Signaling Technology (Boston, USA). Antibody to ITGAV (ab179475, 1:1,000) and antibody to LAMP2a (ab125068, 1:1,000) were purchased from Abcam (Cambridge, UK). ACR (A501033, purity≥98%) was purchased from Sangon Biotech (Shanghai, China). NR1 (IN0240, purity≥98%) was purchased from Solarbio life sciences (Beijing, China). Trx-1 siRNA and rat Trx-1 (NM_053800.3) plasmid were chemically synthesized by Shanghai GeneChem Corporation, Ltd. (Shanghai, China). RIPA Lysis buffer was purchased from Beyotime Biotechnology (Shanghai, China).

### Cell Culture

PC12 cells of the rat pheochromocytoma tumor cell line were purchased from Kunming Institute of Zoology (Kunming, China) and maintained in RPMI1640 medium supplemented with 10% heat-inactivated horse serum, 5% heat-inactivated fetal bovine serum, and antibiotics (100 U/ml penicillin and 100 μg/ml streptomycin) and was maintained at 37°C in a humid atmosphere containing 5% CO_2_.

### Cell Viability Assay

The Cell viability assay was performed to quantify the proliferation of PC12 cells. It was performed using the Cell Counting Kit-8 (Dojindo Laboratories, Kumamoto, Japan). The neuroprotective effects of low doses of NR1 against ACR-induced cell damage was tested on PC12 cells. PC12 cells were seeded in 96-well plates at a density of 5,000 cells per well. Later, PC12 cells were treated with a wide range of concentrations of NR1 (0 mg/ml, 0.01 mg/ml, 0.02 mg/ml, 0.04 mg/ml, and 0.08 mg/ml) or ACR (0 mM, 1 mM, 2 mM, 4 mM, and 8 mM) for 24 h in the 96-well plates. After incubating it for 24 h, CCK-8 reagent was added to each well and again it was incubated for 1 h. The absorbance of each well was read at 450 nm. Three such independent experiments were performed.

### Trx-1 Overexpression Preparation and Cell Transfection

Rat Trx-1 (NM_053800.3) plasmid (4 μg) and lipofectamine™ 2000 (10µl) (per well ratio) were diluted separately in serum-free Opti-MEM to make a final volume of 250 μl. This was gently mixed and incubated for 5 min at room temperature. The diluted plasmid solution and the diluted lipofectamine™ 2000 were mixed together and incubated for 20 min at room temperature. Then, this diluted plasmid/lipofectamine™ 2000 complex was added to the 6-well plates containing PC12 cells. Transfection with the Rat Trx-1 plasmid was allowed to occur for 24 h, and then the cells were stimulated with ACR and NR1. Finally, the cells were harvested for performing different assays.

### Trx-1 siRNA Preparation and Cell Transfection

The sequences of Trx-1 siRNA and negative control siRNA that were used in our study were as follows: Trx-1 siRNA sense: 5’-GUCAAAUGCAUGCCAACAUtt-3’; and anti-sense: 5’-AUGUUGGCAUGCAUUUGACtt-3’. The Trx-1 siRNA was diluted to 20 μM with DEPC (diethyl pyrocarbonate) water.

PC12 cells were plated in 6-well plates at a density of 1×10^5^ cells/well and were allowed to adhere to the plates for 12 h. By using the proportion of 5 μl siRNA and 5 μl lipofectamine™ 2000 per well, the reagents were diluted separately in serum-free Opti-MEM to acquire a final volume of 250μl. It was gently mixed and incubated for 5 min at room temperature. The diluted siRNA solution and the diluted lipofectamine™ 2000 were gently mixed together and incubated for 20 min at room temperature. Then, the diluted siRNA/lipofectamine™ 2000 complex was added to the 6-well plates containing PC12 cells. Transfection of PC12 cells with siRNA was allowed to take place for 24 h, followed by cell stimulation using ACR and NR1. Finally, the cells were harvested for performing different assays.

### Measurement of Intracellular ROS

ROS production was measured using a commercially available intracellular ROS kit (Solarbio, Beijing, China). About 5 × 10^6^ cells were suspended in 1 ml DCFH-DA (10 μmol/L) and were incubated for 20 min at 37°C in the dark. The cells were then washed three times using FBS‐free RPMI1640 medium. Finally, the fluorescence found in the cells was measured using a fluorescent microscope (Olympus CKX53, Tokyo, Japan).

### Western Blot Analysis

Protein lysates was prepared using a solubilizing solution (20 mM Tris–HCl (pH 7.4), 150 mM NaCl, 1% NP-40, 1 mM EDTA, 1 mM phenylmethanesulfonyl fluoride, 1 mM EGTA, 1% Triton X-100, 2.5 mM sodium pyrophosphate, 1 mM Na_3_VO_4_, 1 mM β-glycerol phosphate, and 1 mg/ml leupeptin). The protein concentration found in the cells was determined using a Bio-Rad protein assay reagent (Hercules, CA, USA).

Proteins that were extracted from the cells were separated by using 12% SDS-PAGE (for ITGAV, ATG4B and Cathepsin D, LAMP2a), or 15% SDS-PAGE (for LC3, Trx-1) and were transferred to a polyvinylidene difluoride membrane (Millipore, Billerica, MA, USA). The membrane was then soaked in 10% skimmed milk solution (prepared in phosphate-buffered saline, pH 7.2, containing 0.1% Tween 20) overnight at 4°C. It was then incubated with primary antibody followed by incubation with peroxidase-conjugated anti-mouse or anti-rabbit IgG (KPL, Gaithersburg, MD, USA). The epitope was visualized using an ECL Western blot detection kit (Millipore). Some proteins shared one loading control. Finally, the densitometry analysis was performed using ImageJ software.

### Bioinformatics Informatics

We used transcriptome datasets downloaded from NCBI (GSE29004). Microarray data analysis was performed with Affymetrix GeneChip Operating Software v1.1 (GCOS). A Wilcoxon signed rank test were conducted to detect the expressing gene with “Detection-*p*-value” (P ≤ 0.04). The comparison of gene expression between acrylamide-treated sample and control sample performed with “comparison analysis” tool in GCOS software. The P-value was calculated and used to judge the up-regulated, down-regulated or no-change in gene expression. If P ≤ 0.002, the expression of this gene is up-regulated or down-regulated. Please find details of RNA preparation and microarray hybridization in (Seale et al., 2012). All genes were annotated to the KEGG database by clusterProfiler and enrichplot functions of R software (version 3.5.1) to find important pathway contained specific genes. Spearman’s correlation was computed using cor.test function in R software (version 3.5.1) with gene expression counts to defined genes associated with ITGAV.

### Statistical Analysis

Data were expressed as mean ± SE values. Statistical analysis was performed using SPSS software and GraphPad Prism5 software. A one-way or two-way ANOVA followed by a Bonferroni *post hoc* analysis was performed to identify the differences between the treated groups. A P-value of less than 0.05 was considered statistically significant.

## Results

### NR1 Minimized ACR-Induced Cognitive Dysfunction in Mice

**Figure 1:**
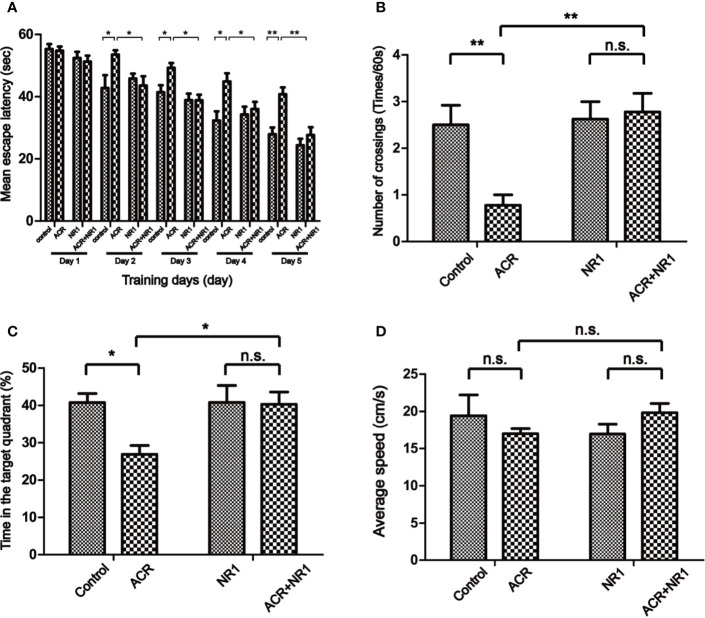
NR1 alleviated ACR-induced cognitive dysfunction in Mice. **(A)** Spatial acquisition phase: the learning curve of escape latency (s) over the five consecutive days of training. Presented was the daily average of four trials (*p < 0.05; **p < 0.01 ACR group compared to control group). **(B)** The number of times the mice crossed the quadrant V. **(C)** The percent of the time each mouse spent in the target quadrant. **(D)** The swimming speed was measured. Each bar represents the mean ± SE (n = 8–9). n.s. (no significance) > 0.05, *P < 0.05, **P < 0.01, statistically significant.

MWM test was performed to evaluate the cognitive dysfunction in mice caused by ACR stimulation. During the spatial acquisition phase, the escape latency for each group had gradually decreased during the five days of training. However, the ACR group was still significantly slower and more sluggish when compared to the control group. It also found that NR1 restored the cognitive dysfunction induced by ACR. From the second day of training, the ACR group spent more amount of time finding the platform when compared to the control group. And, the ACR+NR1 group significantly reduced the escape latency on day 5. Two-way ANOVA revealed a significant NR1 × ACR interaction (F = 4.4, P < 0.05). The effects of ACR (F = 12.63, P < 0.01) and NR1 (F = 13.38, P < 0.01) were considered significant. Bonferroni *post hoc* test showed a significant difference between the control group and the ACR group (P < 0.01), but not in the NR1 group and the ACR+NR1 group (P > 0.05) (**Figure 1A**).

Probe trial performance was an important method to detect spatial memory. Our results have shown that the number of times the mice from the ACR group crossing the quadrant V was significantly less when compared to the control group. Two-way ANOVA revealed a significant NR1 × ACR interaction (F = 6.77, P < 0.05). The effects of ACR (F = 4.74, P < 0.05) and NR1 (F = 8.70, P < 0.01) were considered significant. Bonferroni *post hoc* test showed a significant difference between the control group and the ACR group (P < 0.01), but not in the NR1 group and the ACR+NR1 group (P > 0.05) (**Figure 1B**). In addition, the percentage of time spent in the target quadrant of the ACR group was significantly lower compared to the control group. And there was no significant difference between the NR1 group and the ACR+NR1 group. Two-way ANOVA revealed a significant NR1 × ACR interaction (F = 4.22, P < 0.05). The effects of ACR (F = 4.90, P < 0.05) and NR1 (F = 4.28, P < 0.05) were considered significant. Bonferroni *post hoc* test showed a significant difference between the control group and the ACR group (P < 0.05), but not in the NR1 group and the ACR+NR1 group (P > 0.05) (**Figure 1C**). Besides, we also found that there was no significant difference in the swimming speed between the different experimental groups (P > 0.05) (**Figure 1D**). These results suggested that NR1 could restore ACR-induced cognitive and memory impairment, and this aspect needs further exploration.

### Bioinformatics Analysis for Gene Transcriptional Expression

We have used transcriptome datasets from Seale et al. (2012). Three-week-old male Wistar rat pups were split into two groups and were treated with either acrylamide or saline solution (30 mg/kg) for 21 days. Later the tissues (cerebellum, spinal cord, and sciatic nerve) were harvested and frozen. Two biological replicate samples, each consisting of pooled tissues from 2 rats, were analyzed for each treatment. It found that Pik3r1 was up-regulated. Nr4a1, Nr4a2, Nr4a3, and Fos were down-regulated in the cerebellum after ACR treatment. Pik3r1 was up-regulated. Itgav, Hspa1a/Hspa1b, Myl1, Mcpt8, Bglap, and Mylpf were down-regulated in the spinal cord after ACR treatment. Pik3r1 and Nr1d1 were up-regulated. Vip and Oprk1 were down-regulated in the sciatic nerve after ACR treatment. It also found that ITGAV was down-regulated after treatment with ACR in the spinal cord.

**Figure 2:**
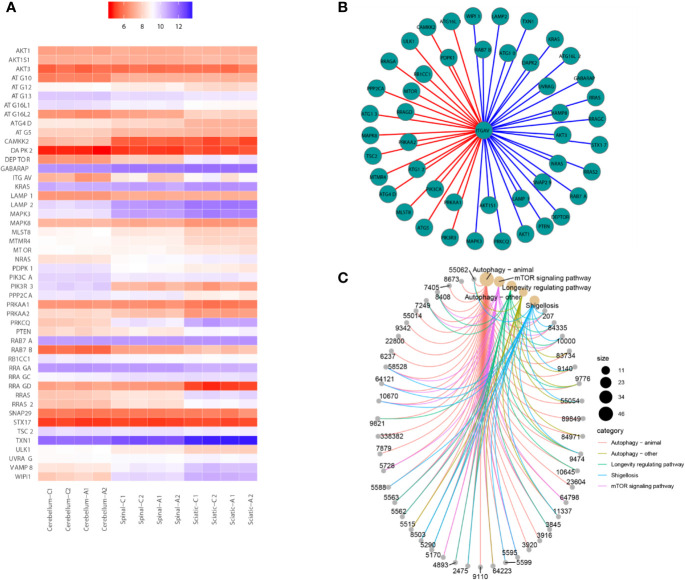
Bioinformatics analysis for gene transcriptional expression. **(A)** Heatmap showed that the expression of genes correlated with ITGAV in 12 samples. **(B)** Correlations between ITGAV and genes in the autophagy pathway or genes in the pathway containing TXN1. **(C)** The genes correlated with ITGAV in the pathway.

Then, all genes were annotated to the KEGG database by clusterProfiler and enrichplot functions of R software (version 3.5.1). All genes of the autophagy pathway containing TXN1 gene were selected, or genes with a significant difference in expression in both the saline and ACR treated groups were selected. Then, Spearman’s correlation of any of the two genes was computed. The gene with a significant correlation (P < 0.05) was found to be the coordinated expression. If two genes resulted in a positive correlation, they were concluded to either be up-regulated or down-regulated. If two genes resulted in a negative correlation, one gene was concluded to be up-regulated while the other was down-regulated. We found that the expression of ITGAV was decreased between saline and ACR treatment groups in **Figure 2A**. **Figure 2B** showed that the genes had significant spearman’s correlations with ITGAV. Besides, using KEGG pathway analysis, **Figure 2C** showed that the KEGG pathway contained genes that were correlated with ITGAV. These genes were found to be grouped under five pathways: Autophagy-animal pathway, Autophagy-other pathway, Longevity regulating pathway, Shigellosis pathway, and mTOR signaling pathway. We also found that the significant genes were clustered in the Autophagy-animal pathway.

### NR1 Suppressed ACR-Induced Autophagy by Regulating ITGAV in the Hippocampus

**Figure 3:**
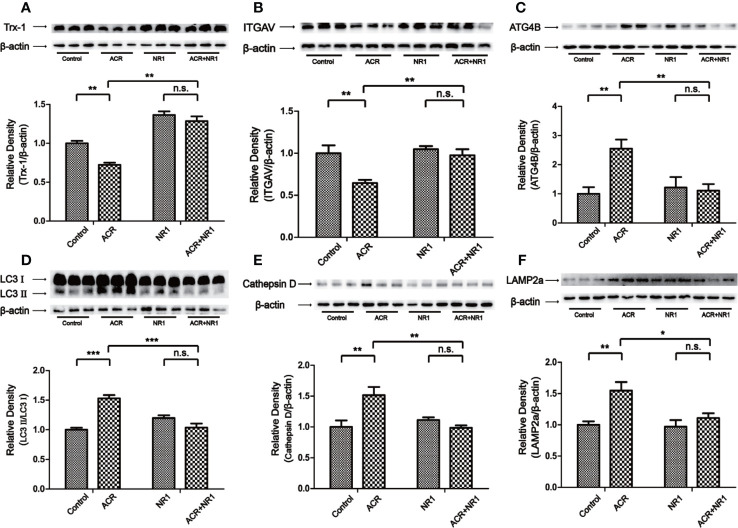
NR1 suppressed ACR-induced autophagy by regulating ITGAV in the hippocampus. **(A)** The expression of Trx-1 in the hippocampus was detected by western blot analysis. NR1 restored the expression of Trx-1. **(B)** The expression of ITGAV in the hippocampus was detected by western blot analysis. NR1 restored the expression of ITGAV. **(C)** The expression of ATG4B in the hippocampus was detected by western blot analysis. NR1 restored the expression of ATG4B. **(D)** The expression of LC3II in the hippocampus was detected by western blot analysis. NR1 restored the expression of LC3II. **(E)** The expression of Cathepsin D in the hippocampus was detected by western blot analysis. NR1 restored the expression of Cathepsin D. **(F)** The expression of LAMP2a in the hippocampus was detected by western blot analysis. NR1 restored the expression of LAMP2a. Each bar represents the mean ± SE (*n* = 6). n.s. (no significance) >0.05, *P < 0.05, **P < 0.01, ***P < 0.001, statistically significant.

To reveal the protective mechanism of NR1, Trx-1 had been expressed in the hippocampus of mice. The results also showed that the expression of Trx-1 was lower in the ACR group when compared to the control group. And NR1 was identified to restore the expression of Trx-1 in the ACR+NR1 group. Two-way ANOVA revealed a significant NR1 × ACR interaction (F = 5.13, P < 0.05). The effects of ACR (F = 16.48, P < 0.001) and NR1 (F = 112.2, P < 0.001) were considered significant. Bonferroni *post hoc* test showed a significant difference between the control group and the ACR group (P < 0.01), but not in the NR1 group and the ACR+NR1 group (P > 0.05) (**Figure 3A**). Besides, we also investigated the expression of ITGAV. Two-way ANOVA revealed a significant NR1 × ACR interaction (F = 4.77, P < 0.05). The effects of ACR (F = 10.70, P < 0.001) and NR1 (F = 8.46, P < 0.001) were considered significant. Bonferroni *post hoc* test showed a significant difference between the control group and the ACR group (P < 0.01), but not in the NR1 group and the ACR+NR1 group (P > 0.05) (**Figure 3B**). This result was found to be consistent with the results obtained after bioinformatics analysis. We also found that the expression levels of ATG4B, LC3II, Cathepsin D, and LAMP2a were elevated, however, NR1 restored the expression of these molecules. Two-way ANOVA revealed a significant NR1 × ACR interaction (F = 8.39, P < 0.01). The effects of ACR (F = 6.38, P < 0.05) and NR1 (F = 4.56, P < 0.05) were considered significant. Bonferroni *post hoc* test showed a significant difference between the control group and the ACR group (P < 0.01), but not in the NR1 group and the ACR+NR1 group (P > 0.05) (**Figure 3C**). Two-way ANOVA revealed a significant NR1 × ACR interaction (F = 42.88, P < 0.001). The effects of ACR (F = 12.12, P < 0.01) and NR1 (F = 7.92, P < 0.05) were considered significant. Bonferroni *post hoc* test showed a significant difference between the control group and the ACR group (P < 0.001), but not in the NR1 group and the ACR+NR1 group (P > 0.05) (**Figure 3D**). Two-way ANOVA revealed a significant NR1 × ACR interaction (F = 13.12, P < 0.01). The effects of ACR (F = 4.79, P < 0.01) and NR1 (F = 5.55, P < 0.05) were considered significant. Bonferroni *post hoc* test showed a significant difference between the control group and the ACR group (P < 0.01), but not in the NR1 group and the ACR+NR1 group (P > 0.05) (**Figure 3E**). Two-way ANOVA revealed a significant NR1 × ACR interaction (F = 4.37, P < 0.05). The effects of ACR (F = 12.20, P < 0.01) and NR1 (F = 5.69, P < 0.05) were considered significant. Bonferroni *post hoc* test showed a significant difference between the control group and the ACR group (P < 0.01), but not in the NR1 group and the ACR+NR1 group (P > 0.05) (**Figure 3F**).

### Effects of NR1 or ACR on PC12 Cells Viability and Trx-1 Expression

**Figure 4:**
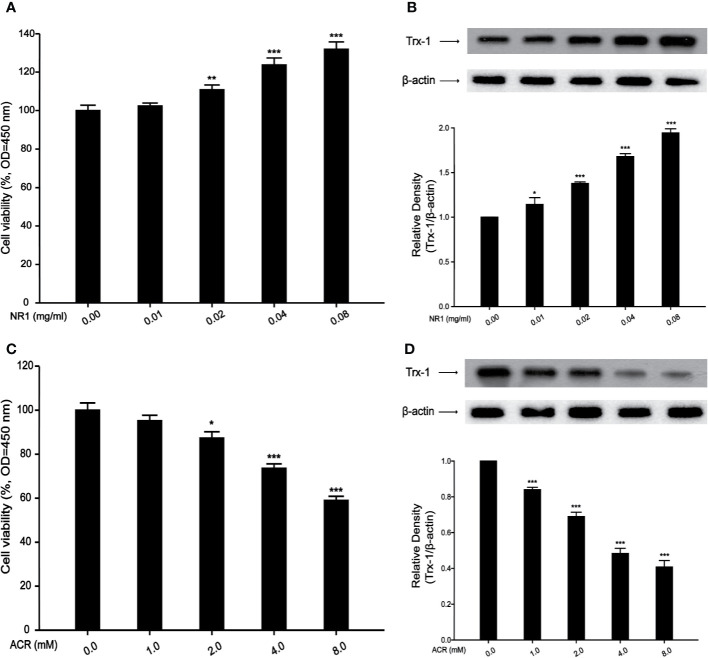
Effects of NR1 or ACR on PC12 cells viability and Trx-1 expression. **(A)** Effects of NR1 on PC12 cells viability. **(B)** Effects of NR1 on PC12 cells Trx-1 expression. **(C)** Effects of ACR on PC12 cells viability. **(D)** Effects of ACR on PC12 cells Trx-1 expression. Each bar represents the mean ± SE (n = 3 independent experiments). n.s. (no significance) > 0.05, *P < 0.05, **P < 0.01, ***P < 0.001, statistically significant.

An *in vitro* study was undertaken, to explore the mechanism of action of ACR and NR1 on PC12 cells. Hong et al. proved in their study that ACR induced ROS in BRL-3A cells (Hong et al., 2019). Recent studies have also found that *Panax notoginseng* saponins decreased the level of ROS. However, the mechanism of ACR and NR1 on how it affects the ROS pathway in the nervous system remains unclear. To detect the dose–response, PC12 cells were treated with NR1 at concentrations ranging from 0.01 to 0.08 mg/ml for 24 h. PC12 cells were also treated with ACR at concentrations ranging from 1 to 8 mM for 24 h. The cell viability test was performed using a CCK-8 assay. It was found that both the cell viability as well as the Trx-1 expression levels had increased following the treatment of the cells with increasing concentrations of NR1 (0, 0.01, 0.02, 0.04, and 0.08 mg/ml) (**Figures 4A, B**). And we also found that cell viability and Trx-1 expression levels were decreased by treatment with increasing concentrations of ACR (0, 1, 2, 4, and 8 mM) (**Figures 4C, D**). Here, in this experiment, the concentration levels of NR1 and ACR were optimized and narrowed down to 0.04 mg/ml NR1 and 4 mM ACR.

### NR1 Suppressed ACR-Induced ROS in PC12 Cells

**Figure 5:**
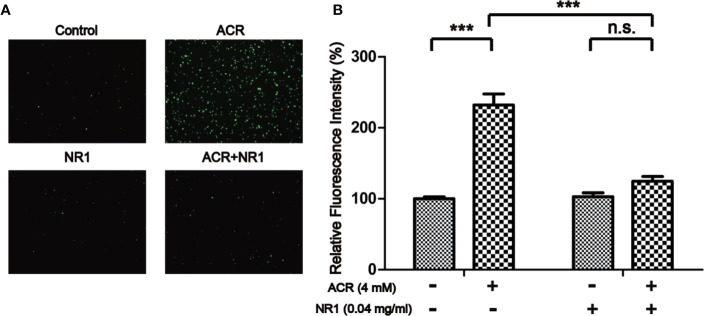
NR1 suppressed ACR-induced ROS in PC12 cells. **(A)** Representative photographs of ROS staining. **(B)** Quantitative analysis of the DCFH-DA fluorescence intensity. Each bar represents the mean ± SE (n = 3 independent experiments). n.s. (no significance) > 0.05, ***P < 0.001, statistically significant (Magnification×100).

The probe DCFH-DA could be oxidized to form a fluorescent compound called DCF. This indirectly indicated the levels of ROS in PC12 cells. As shown in **Figure 5**, the fluorescence intensity of DCFH-DA was evidently strengthened by ACR, indicating an excessive amount of accumulation of ROS. The results have also shown that NR1 could decrease the intracellular ROS levels and halt the oxidative stress triggered by ACR. Two-way ANOVA revealed a significant NR1 × ACR interaction (F =37.46, P < 0.001). The effects of ACR (F = 73.16, P < 0.05) and NR1 (F = 33.70, P < 0.001) were considered significant. Bonferroni *post hoc* test showed a significant difference between the control group and the ACR group (P < 0.001), but not in the NR1 group and the ACR+NR1 group (P > 0.05) (**Figure 5**).

### NR1 Suppressed ACR-Induced Autophagy by Regulating ITGAV in PC12 Cells

**Figure 6:**
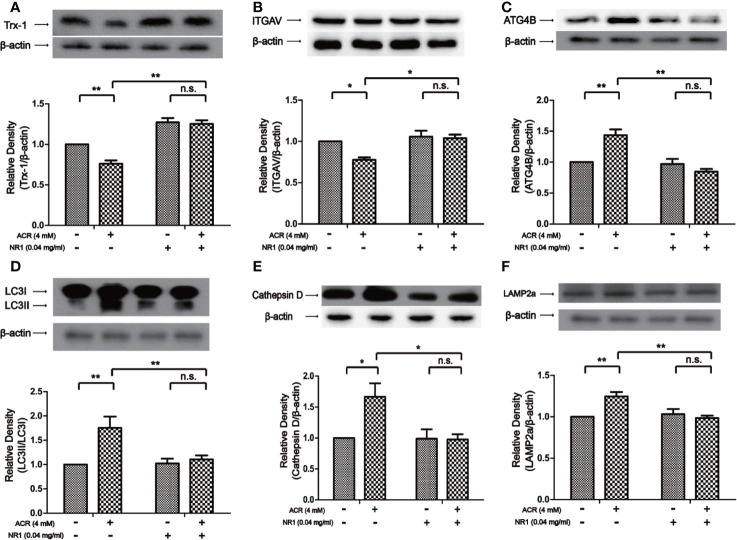
NR1 suppressed ACR-induced autophagy by regulating ITGAV in PC12 cells. **(A)** The expression of Trx-1 in PC12 cells was detected by western blot analysis. NR1 restored the expression of Trx-1. **(B)** The expression of ITGAV in PC12 cells was detected by western blot analysis. NR1 restored the expression of ITGAV. **(C)** The expression of ATG4B in PC12 cells was detected by western blot analysis. NR1 restored the expression of ATG4B. **(D)** The expression of LC3II in PC12 cells was detected by western blot analysis. NR1 restored the expression of LC3II. **(E)** The expression of Cathepsin D in PC12 cells was detected by western blot analysis. NR1 restored the expression of Cathepsin D. **(F)** The expression of LAMP2a in PC12 cells was detected by western blot analysis. NR1 restored the expression of LAMP2a. Each bar represents the mean ± SE (n = 3 independent experiments). n.s. (no significance) > 0.05, *P < 0.05, **P < 0.01, statistically significant. Trx-1 and LAMP2a shared one loading control.

Using bioinformatics analysis, we have established that the mRNA level of ITGAV was reduced after ACR administration when compared to the control group. Mridu had reported that ITGAV plays an important role in regulating autophagy in B-cell (Acharya et al., 2016). In our results, we have shown that NR1 inhibited the cytotoxicity triggered by ACR. We have also shown that the decrease in Trx-1 expression triggered by ACR was repressed by NR1. Two-way ANOVA revealed a significant NR1 × ACR interaction (F = 7.65, P < 0.05). The effects of ACR (F = 10.52, P < 0.05) and NR1 (F = 92.52, P < 0.001) were considered significant. Bonferroni *post hoc* test showed a significant difference between the control group and the ACR group (P < 0.01), but not in the NR1 group and the ACR+NR1 group (P > 0.05) (**Figure 6A**). As described above, the expression of Trx-1 was associated with ITGAV. Therefore, we found that the expression of ITGAV had decreased. NR1 restored the expression levels of ITGAV. Two-way ANOVA revealed a significant NR1 × ACR interaction (F = 5.32, P < 0.05). The effects of ACR (F = 7.46, P < 0.05) and NR1 (F = 13.26, P < 0.01) were considered significant. Bonferroni *post hoc* test showed a significant difference between the control group and the ACR group (P < 0.05), but not in the NR1 group and the ACR+NR1 group (P > 0.05) (**Figure 6B**). Besides, we investigated the autophagy pathway associated proteins ATG4B, LC3II, Cathepsin D, and LAMP2a. We found that the increase in the levels of ATG4B, LC3II, Cathepsin D, and LAMP2a expression induced by ACR were repressed by NR1. Two-way ANOVA revealed a significant NR1 × ACR interaction (F = 18.53, P < 0.01). The effects of ACR (F = 5.80, P < 0.05) and NR1 (F = 22.72, P < 0.01) were considered significant. Bonferroni *post hoc* test showed a significant difference between the control group and the ACR group (P < 0.01), but not in the NR1 group and the ACR+NR1 group (P > 0.05) (**Figure 6C**). Two-way ANOVA revealed a significant NR1 × ACR interaction (F = 6.50, P < 0.05). The effects of ACR (F = 10.12, P < 0.05) and NR1 (F = 5.59, P < 0.05) were considered significant. Bonferroni *post hoc* test showed a significant difference between the control group and the ACR group (P < 0.01), but not in the NR1 group and the ACR+NR1 group (P > 0.05) (**Figure 6D**). Two-way ANOVA revealed a significant NR1 × ACR interaction (F = 6.02, P < 0.05). The effects of ACR (F = 5.55, P < 0.05) and NR1 (F = 6.38, P < 0.05) were considered significant. Bonferroni *post hoc* test showed a significant difference between the control group and the ACR group (P < 0.05), but not in the NR1 group and the ACR+NR1 group (P > 0.05) (**Figure 6E**). Two-way ANOVA revealed a significant NR1 × ACR interaction (F =11.94, P < 0.01). The effects of ACR (F = 5.38, P < 0.05) and NR1 (F = 7.27, P < 0.05) were considered significant. Bonferroni *post hoc* test showed a significant difference between the control group and the ACR group (P < 0.01), but not in the NR1 group and the ACR+NR1 group (P > 0.05) (**Figure 6F**). These data suggested that NR1 can resist ACR-induced autophagy in PC12 cells.

### Trx-1 Overexpression Attenuated ACR-Induced Autophagy by Regulating ITGAV in PC12 Cells

**Figure 7:**
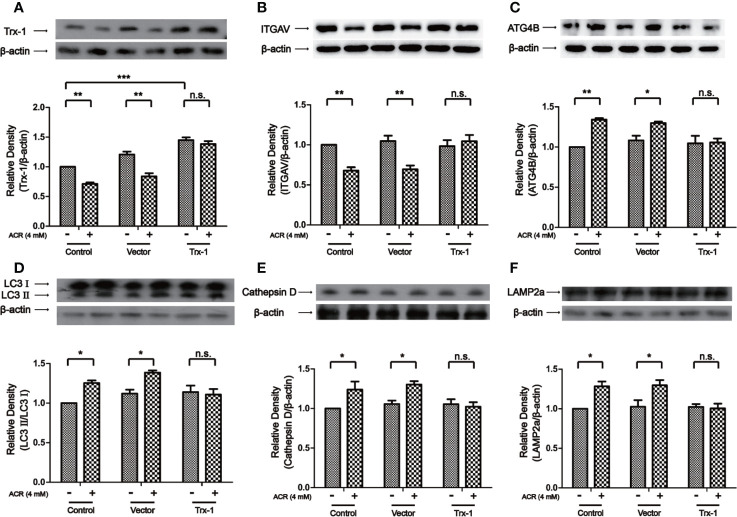
Trx-1 overexpression attenuated ACR-induced autophagy by regulating ITGAV in PC12 cells. **(A)** The expression of Trx-1 in PC12 cells was detected by western blot analysis in PC12 cells. Trx-1 overexpression restored the expression of Trx-1. **(B)** The expression of ITGAV in PC12 cells was detected by western blot analysis. Trx-1 overexpression restored the expression of ITGAV. **(C)** The expression of ATG4B in PC12 cells was detected by western blot analysis. Trx-1 overexpression restored the expression of ATG4B. **(D)** The expression of LC3II in PC12 cells was detected by western blot analysis. Trx-1 overexpression restored the expression of LC3II. **(E)** The expression of Cathepsin D in PC12 cells was detected by western blot analysis. Trx-1 overexpression restored the expression of Cathepsin D. **(F)** The expression of LAMP2a in PC12 cells was detected by western blot analysis. Trx-1 overexpression restored the expression of LAMP2a. Each bar represents the mean ± SE (n = 3 independent experiments). n.s. (no significance) > 0.05, *P < 0.05, **P < 0.01, ***P < 0.001, statistically significant.

Our results have validated that NR1 can resist ACR-induced cell apoptosis, however, the detailed mechanism is still unknown. Previous studies had proven that NR1 could increase the expression of Trx-1 and thereby protect the neurons (Wang et al., 2014; Zeng et al., 2015). Therefore, we hypothesized that the overexpression of the protein Trx-1 could probably suppress ACR-induced autophagy. Next, we investigated the effects of overexpression of Trx-1 on ACR-induced autophagy in PC12 cells which were transfected with rat Trx-1 plasmid. As shown in **Figure 7A**, Trx-1 was overexpressed in the PC12 cells which was transfected with rat Trx-1 plasmid and it reversed the decline of Trx-1 induced by ACR. Two-way ANOVA revealed a significant Trx-1 overexpression × ACR interaction (F = 7.18, P < 0.01). The effects of ACR (F = 50.27, P < 0.001) and Trx-1 overexpression (F = 95.54, P < 0.001) were considered significant. Bonferroni *post hoc* test showed a significant difference between the control group and the ACR group (P < 0.01), but not in the Trx-1 overexpression group and the ACR+Trx-1 overexpression group (P > 0.05) (**Figure 7A**). Apart from that, overexpression of Trx-1 restored the expression of ITGAV (**Figure 7B**) and the autophagy pathway associated proteins—ATG4B, LC3II, Cathepsin D, and LAMP2a (**Figures 7C–F**). Two-way ANOVA revealed a significant Trx-1 overexpression × ACR interaction (F = 7.81, P < 0.01). The effects of ACR (F = 18.33, P < 0.01) and Trx-1 overexpression (F = 5.07, P < 0.05) were considered significant. Bonferroni *post hoc* test showed a significant difference between the control group and the ACR group (P < 0.01), but not in the Trx-1 overexpression group and the ACR+Trx-1 overexpression group (P > 0.05) (**Figure 7B**). Two-way ANOVA revealed a significant Trx-1 overexpression × ACR interaction (F = 5.24, P < 0.05). The effects of ACR (F = 20.47, P < 0.001) and Trx-1 overexpression (F = 4.38, P < 0.05) were considered significant. Bonferroni *post hoc* test showed a significant difference between the control group and the ACR group (P < 0.01), but not in the Trx-1 overexpression group and the ACR+Trx-1 overexpression group (**Figure 7C**). Two-way ANOVA revealed a significant Trx-1 overexpression × ACR interaction (F = 5.29, P < 0.05). The effects of ACR (F = 14.96, P < 0.01) and Trx-1 overexpression (F = 4.07, P < 0.05) were considered significant. Bonferroni *post hoc* test showed a significant difference between the control group and the ACR group (P < 0.05), but not in the Trx-1 overexpression group and the ACR+Trx-1 overexpression group (**Figure 7D**). Two-way ANOVA revealed a significant Trx-1 overexpression × ACR interaction (F = 4.96, P < 0.05). The effects of ACR (F = 13.78, P < 0.01) and Trx-1 overexpression (F = 3.98, P < 0.05) were considered significant. Bonferroni *post hoc* test showed a significant difference between the control group and the ACR group (P < 0.05), but not in the Trx-1 overexpression group and the ACR+Trx-1 overexpression group (P > 0.05) (**Figure 7E**). Two-way ANOVA revealed a significant Trx-1 overexpression × ACR interaction (F = 4.42, P < 0.05). The effects of ACR (F = 14.51, P < 0.01) and Trx-1 overexpression (F = 3.89, P < 0.05) were considered significant. Bonferroni *post hoc* test showed a significant difference between the control group and the ACR group (P < 0.05), but not in the Trx-1 overexpression group and the ACR+Trx-1 overexpression group (P > 0.05) (**Figure 7F**). These results suggested that overexpression of Trx-1 attenuated the ACR-induced autophagy by restoring the expression of ITGAV, ATG4B, LC3II, Cathepsin D, and LAMP2a.

### Trx-1 siRNA Aggravated ACR-Induced Autophagy by Regulating ITGAV in PC12 Cells

**Figure 8:**
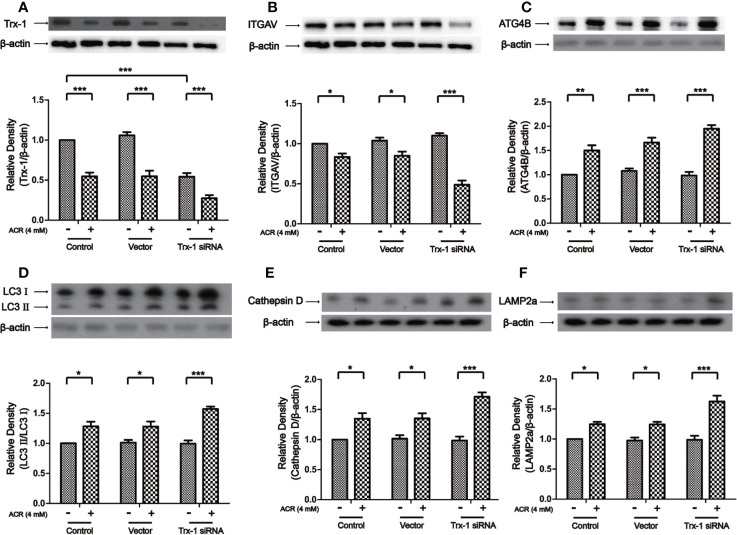
Trx-1 siRNA aggravated ACR-induced autophagy by regulating ITGAV in PC12 cells. **(A)** The expression of Trx-1 in PC12 cells was detected by western blot analysis. Trx-1 expression has further decreased after Trx-1 siRNA treatment on PC12 cells followed by ACR treatment. **(B)** The expression of ITGAV in PC12 cells was detected by western blot analysis. The expression of ITGAV has further decreased after Trx-1 siRNA treatment on PC12 cells followed by ACR treatment. **(C)** The expression of ATG4B in PC12 cells was detected by western blot analysis. The expression of ATG4B has further increased after Trx-1 siRNA treatment on PC12 cells followed by ACR treatment. **(D)** The expression of LC3II in PC12 cells was detected by western blot analysis. The expression of LC3II has further increased after Trx-1 siRNA treatment on PC12 cells followed by ACR treatment. **(E)** The expression of Cathepsin D in PC12 cells was detected by western blot analysis. The expression of Cathepsin D has further increased after Trx-1 siRNA treatment on PC12 cells followed by ACR treatment. **(F)** The expression of LAMP2a in PC12 cells was detected by western blot analysis. The expression of LAMP2a has further increased after Trx-1 siRNA treatment on PC12 cells followed by ACR treatment. Each bar represents the mean ± SE (n = 3 independent experiments). n.s. (no significance) > 0.05, *P < 0.05, **P < 0.01, ***P < 0.001, statistically significant. Trx-1 and ITGAV shared one loading control. Cathepsin D and LAMP2a shared one loading control.

Then, by using Trx-1 siRNA on ACR-induced autophagy, we examined the effects of downregulation of Trx-1 expression. The results obtained showed that Trx-1 expression had declined due to the presence of Trx-1 siRNA in PC12 cells. Trx-1 expression has further decreased by the presence of ACR along with the presence of Trx-1 siRNA in PC12 cells. Two-way ANOVA revealed a significant Trx-1 siRNA × ACR interaction (F = 3.90, P < 0.05). The effects of ACR (F = 121.60, P < 0.001) and Trx-1 siRNA (F = 46.66, P < 0.001) were considered significant. Bonferroni *post hoc* test showed a significant difference between the control group and the ACR group (P < 0.001), and there was also a significant difference between the Trx-1 siRNA group and the ACR+ Trx-1 siRNA group (P < 0.001) (**Figure 8A**). Besides, the expression of ITGAV has further declined after Trx-1 siRNA treatment on PC12 cells followed by ACR treatment. Two-way ANOVA revealed a significant Trx-1 siRNA × ACR interaction (F = 18.81, P < 0.001). The effects of ACR (F = 92.51, P < 0.001) and Trx-1 siRNA (F = 7.61, P < 0.01) were considered significant. Bonferroni *post hoc* test showed a significant difference between the control group and the ACR group (P < 0.05), and there was also a significant difference between the Trx-1 siRNA group and the ACR+ Trx-1 siRNA group (P < 0.001) (**Figure 8B**). More importantly, the expression of ATG4B, LC3II, Cathepsin D, and LAMP2a have further increased after Trx-1 siRNA treatment on PC12 cells followed by ACR treatment. Two-way ANOVA revealed a significant Trx-1 siRNA × ACR interaction (F = 5.18, P < 0.05). The effects of ACR (F = 120.06, P < 0.001) and Trx-1 siRNA (F = 3.96, P < 0.05) were considered significant. Bonferroni *post hoc* test showed a significant difference between the control group and the ACR group (P < 0.01), and there was also a significant difference between the Trx-1 siRNA group and the ACR+ Trx-1 siRNA group (P < 0.001) (**Figure 8C**). Two-way ANOVA revealed a significant Trx-1 siRNA × ACR interaction (F = 4.64, P < 0.05). The effects of ACR (F = 68.85, P < 0.001) and Trx-1 siRNA (F = 3.98, P < 0.05) were considered significant. Bonferroni *post hoc* test showed a significant difference between the control group and the ACR group (P < 0.05), and there was also a significant difference between the Trx-1 siRNA group and the ACR+ Trx-1 siRNA group (P < 0.001) (**Figure 8D**). Two-way ANOVA revealed a significant Trx-1 siRNA × ACR interaction (F = 5.24, P < 0.05). The effects of ACR (F = 69.72, P < 0.001) and Trx-1 siRNA (F = 4.13, P < 0.05) were considered significant. Bonferroni *post hoc* test showed a significant difference between the control group and the ACR group (P < 0.05), and there was also a significant difference between the Trx-1 siRNA group and the ACR+ Trx-1 siRNA group (P < 0.001) (**Figure 8E**). Two-way ANOVA revealed a significant Trx-1 siRNA × ACR interaction (F = 7.43, P < 0.01). The effects of ACR (F = 67.58, P < 0.001) and Trx-1 siRNA (F = 7.42, P < 0.01) were considered significant. Bonferroni *post hoc* test showed a significant difference between the control group and the ACR group (P < 0.05), and there was also a significant difference between the Trx-1 siRNA group and the ACR+ Trx-1 siRNA group (P < 0.001) (**Figure 8F**). These data suggested that Trx-1 siRNA treatment promoted ACR-induced autophagy in PC12 cells by inhibiting ITGAV and inducing of ATG4B, LC3II, Cathepsin D, and LAMP2a (**Figures 8B–F**).

## Discussion

According to laboratory evidence, ACR has been classified as a possible human carcinogen. It has also been proved that dietary acrylamide intake causes esophageal, gastric, and colorectal cancer (Liu R. et al., 2019). Besides, lots of studies have proved that neurotoxicity of acrylamide in tissues and organs has been confirmed at the animal levels (Song et al., 2008; Tomaszewska et al., 2014). In our present study, using bioinformatics analysis, we have found that the mRNA level of ITGAV was decreased, and there was a correlation between ITGAV and TXN1. Besides, we also found that ITGAV played an important role in autophagy.

Our results have suggested a new mechanism for the regulation of autophagy by regulating ITGAV, which was mediated by Trx-1. Previous studies have proven that integrin was closely involved in the autophagy pathway targeting LC3 and ATG5. Besides, Mridu had already shown that ITGAV promotes the recruitment of the autophagy component LC3 (Acharya et al., 2016). Here, using bioinformatics analysis, we have shown that there was an important link between Trx-1 and ITGAV. Therefore, Trx-1 was an important regulator of autophagy by regulating ITGAV.

Notoginsenoside R1 is a plant extract, purified from *Panax notoginseng* and can be used in the treatment of various types of diseases, such as cerebral ischemia (Xie et al., 2018) and cardiovascular diseases (Yang et al., 2014). Our results have shown that NR1 can induce the expression of Trx-1. Trx-1 played a vital role in various diseases, including ischemic stroke (Qi et al., 2015), morphine addiction (Zeng et al., 2020), and Parkinson’s disease (Zeng et al., 2014). Besides, Wang et al. proved that NR1 alleviated ROS which was induced by high glucose present in RSC96 cells (Wang et al., 2019). And Zhang et al. suggested that NR1 inhibited apoptosis in smooth muscle cells through the ROS pathway (Zhang and Wang, 2006). By pretreatment of the cells with H_2_O_2_, the expression of ITGAV was found to be reduced. Murata et al. (2013) highlighted the relationship between ITGAV and ROS. Previous studies have reported that autophagy exerted a pleiotropic action on multiple cell functions *via* the generation of ROS (Sun et al., 2019; Zhang et al., 2019). It was also possible for NR1 to alter the nerve cell function by regulating Trx-1 expression and thereby the expression of ITGAV was controlled *via* suppression of ROS generation. N-acetylcysteine inhibited ACR-induced ROS in PC12 cells by regulating the mitogen-activated protein kinases (MAPKs) pathway (Pan et al., 2018). Zhang et al. (2017) also proved that the cytotoxicity induced by acrylamide was through the production of ROS. Also, Singh et al. (2017) suggested that ROS-induced activation of autophagy resulted in neurotransmission dysfunction and neurodegeneration in adult rats. Therefore, sum total of these studies have shown that the key mechanism of ACR-induced neurotoxicity was an increase in the levels of ROS. Our results suggested that NR1 inhibited the level of ROS by triggering an increase of Trx-1, and this Trx-1 helped to regulate autophagy by regulating ITGAV. Furthermore, we also found that NR1 resisted ACR-induced spatial and cognitive impairment in mice. In summary, ACR induced oxidative damage *via* ROS generation. ROS production induced the decrease of ITGAV expression, which led to autophagy. Finally, it led to cognitive dysfunction. NR1 could inhibit ROS production by upregulating Trx-1 expression. Although we demonstrated the neuroprotective effects of Trx-1, the specific mechanism of how TRX affected ITGAV needed further study. It was also worth exploring the effects of knockdown or overexpression of ITGAV in PC12 cells after ACR administration. Therefore, NR1 can reduce the accumulation of ROS and represent a promising new avenue for the development of novel treatments for the ACR-induced neurotoxicity.

## Conclusion

To summarize, ACR aggravates autophagy in PC12 cells by knocking down the expression of Trx-1, on the contrary, overexpression of Trx-1 and NR1 inhibits autophagy induced by ACR. We have also found that ITGAV was an intermediate node linking Trx-1 and the autophagy pathway. Hence, we can conclude by suggesting that NR1 may be a potential drug for the treatment of acrylamide-induced neurotoxicity.

## Data Availability Statement

All datasets presented in this study are included in the article/supplementary material.

## Ethics Statement

The animal study was reviewed and approved by animal ethics council of Southwest Medical University.

## Author Contributions

XL was responsible for the study concept and design. WW, LH, and YH did the experiments. WW and LH drafted the manuscript. XL, FZ, and ET provided a critical revision of the manuscript for important intellectual content. All authors contributed to the article and approved the submitted version.

## Funding

This study was financially supported by grants from the Science and Technology Strategic Cooperation Project of the Luzhou People’s Government and Southwest Medical University (No. 2019LZXNYDJ34) and the undergraduate innovation and entrepreneurship training program (S201910727096). This work was also supported by grants from the Science and Technology Planning Project of Sichuan Province, China (Grant No. 2013SZZ001).

## Conflict of Interest

The authors declare that the research was conducted in the absence of any commercial or financial relationships that could be construed as a potential conflict of interest.
